# Design and Simulation of an Ultra-Low-Power Hydrogen Sulfide Gas Sensor with a Cantilever Structure

**DOI:** 10.3390/mi15030295

**Published:** 2024-02-21

**Authors:** Xin Tian, Jifang Tao, Maosen Xu, Yuzhe Lin, Jia Zhao

**Affiliations:** 1 Key Laboratory of Laser and Infrared System Ministry of Education, Shandong University, Qingdao 266237, China; tianxin1012@mail.sdu.edu.cn; 2 School of Information Science and Engineering, Shandong University, Qingdao 266237, China; linyz@sdu.edu.cn (Y.L.); zhaojia@sdu.edu.cn (J.Z.); 3 School of Electrical Engineering and Automation, Shandong University of Science and Technology, Qingdao 266590, China; xumaosenld@163.com

**Keywords:** ultra-low power, gas sensor, straight beam, corrugated beam, size effect

## Abstract

Metal oxide gas sensors usually require a few tens of milliwatts of power consumption to operate at high temperature, which limits their application in mobile and portable devices. Here, we proposed a cantilever structure to build an ultra-low power gas sensor for hydrogen sulfide gas detection. By employing a nano-film size effect to reduce the thermal conductivity of the material, and self-heated corrugation configuration, the power consumption of the gas sensor is significantly reduced. Through numerical analysis and finite element simulation, two different gas sensors were designed and the power consumption and stress distribution were analyzed and optimized. Under the operating temperature of 200 °C, only 0.27 mW power is consumed, the stress value is less than 250 MPa and the displacement is a few hundred of nanometers. The results serve as a guide and reference for ultra-low power MEMS device designs.

## 1. Introduction

The metal oxide gas sensors based on micro-electromechanical systems (MEMSs) possess the advantages of cost-effectiveness, miniaturization, and facile manufacturing, thereby exhibiting extensive prospects for applications [1,2,3,4]. However, these devices necessitate a substantial power consumption (a few tens of milliwatts), as metal oxide sensors require a high temperature for operation. This limitation hampers their applicability in mobile and portable systems [5,6,7].

The structure of MEMS gas sensors usually includes a suspension, dielectric layers, a sensing powder material and a micro-heater. When the current passes through the heater electrode, part of the joule heat generated by the resistance is used to heat the micro-hotplate chip, and the other part is dissipated to the surrounding environment in the way of conduction, convection and radiation [8]. Reducing the size of the micro-heater is the most effective way to reduce overall power consumption [9]. Prajapati et al. report an ultra-low-power platform with nanoheater of size 4 μm × 100 nm, which consumes ∼1.8 mW power when operated continuously at 300 °C [10]. Courbat et al. designed a small (15 × 15 μm) micro-hotplate, and the power consumption is 6 mW at 300 °C [11].The power consumption of the micro-hotplate can also be reduced by decreasing both the number and width of the cantilevers. Xu et al. demonstrated that the micro-hotplate with only two beams exhibits a further reduction of approximately 44% in heat loss compared to the micro-hotplate with four beams, when both reach the same heating area temperature [12]. In addition, the reduction in power consumption can also be achieved by minimizing thermal conductivity. The heat transfer law of nanosensor exhibits a distinct micro-scale effect when it is refined, deviating from the conventional scale [13]. Xu et al. proposed four devices for measuring microthermal conductivity (μTCM) of silicon oxide, polysilicon, and aluminum films, and the experimental results showed that the measured thermal conductivity was significantly lower than the corresponding bulk values [14].

Over the past few decades, the development of silicon micromachining technology has enabled the manufacture of micro-cantilever beams, and the development of advanced signal reading technology has made micro-cantilever beams widely used in chemical, biological, microbial and pharmaceutical fields. In the field of gas detection, Finot et al. [15] utilized silicon nitride and silicon oxide microcantilever beams to achieve trace detection of hydrofluoric acid gas within a detection range of 0.23–13 ppm, while also investigating the underlying detection mechanism. The detection of drugs and small molecules by the cantilever sensor holds significant importance in drug development and elucidating the mechanism of action. McKendry et al. [16] investigated the interaction between vancomycin and peptides on a gold-plated surface of a micro-cantilever beam, which was modified with an anti-vancomycin polypeptide, leading to successful detection of vancomycin with a minimum limit of 10 nmol/L. Mao et al. [17] describe a fabrication method suitable for microfluidic applications in which the SU-8 cantilever is vertically distributed in a closed fluid channel. The primary objective of this study is to focus on the design and simulation of the cantilever beam gas sensor, with a specific emphasis on optimizing the shape, thickness, and dimensions of the cantilever in order to achieve the desired minimum power consumption. Burge et al. first proposed the potential application of silver films as highly responsive sensors for detecting trace amounts of H_2_S [18]. Silver-based MEMS gas sensors capable of rapidly detecting hydrogen sulfide at high temperatures have been demonstrated [19]. In this paper, a low-power hydrogen sulfide gas sensor that operates within the ultra-low power range (<0.3 mW at 200 °C) has been demonstrated. The corrugated cantilever structure of heater in this paper is also useful for other gas sensors applications, which serve as a guide and reference for ultra-low power MEMS device designs.

## 2. Design and Materials

**Figure 1:**
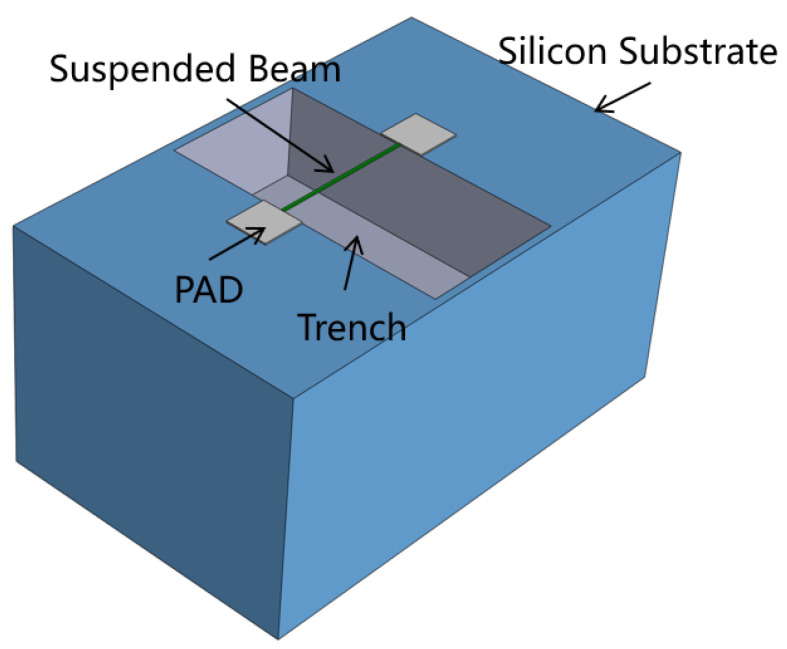
Design concept of suspended beam.

According to the theory of heat, the heat transfer paths of micro-hotplate include heat conduction, heat convection and heat radiation. The total heat flow can be expressed as follows [8]:

(1) $$Q_{total}=Q_{cond}+Q_{conv}+Q_{rad}$$

where $Q_{cond}$ is the heat conduction loss, $Q_{conv}$ is air thermal convection loss, and $Q_{rad}$ is the thermal radiation loss. The power consumption of micro-hotplate is primarily determined by the heat loss from the supporting substrate. “Suspended beam” designs are widely adopted to minimize thermal contact between the heater and substrate, as shown in Figure 1. Most of the heat losses are caused by heat conduction through the beam and the surrounding air. Operation of the micro-hotplate is usually in the few hundred degrees, which makes radiated losses low and, to an extent, negligible [20]. Assuming that the center temperature of the beam is at its maximum and the anchor point of the beam remains at room temperature, the power loss resulting from conduction within the beam is calculated as follows:

(2) $$P_{beam}=\frac{8k_{eff}Wt}{L}\Delta T$$

where $k_{eff}$ is the thermal conductivity of the beam stack, *W* is the beam width and *L* is the beam length. The power lost to the surrounding air is approximated to be [21]

(3) $$P_{air}=\frac{\pi k_{air}L}{In(L/W)}\Delta T$$

The total power consumption is, therefore, modeled as

(4) $$P=P_{air}+P_{beam}=\Delta T\left(\frac{\pi k_{air}L}{In(L/W)}+\frac{8_{keff}Wt}{L}\right)$$

Heat loss is closely related to the thermal conductivity of material, the thickness, length and width of the beam.

One of the primary challenges in designing MEMS gas sensors lies in achieving exceptional thermomechanical stability. Stacked structures are susceptible to heat-induced stress during both manufacturing and operation stages. Thermal stress ($\sigma_{th}$) significantly contributes to stress accumulation within the film layer, and can be expressed using [22]

(5) $$\sigma_{th}=E\alpha \left(T-T_{0}\right)$$

where *E* is the Young modulus, α is the coefficient of thermal expansion, and $T-T_{0}$ is the temperature difference leading to the rise in stress. When choosing materials, it is preferable to select those with internal stresses that can mutually offset each other and the low coefficient of thermal expansion.

**Figure 2:**
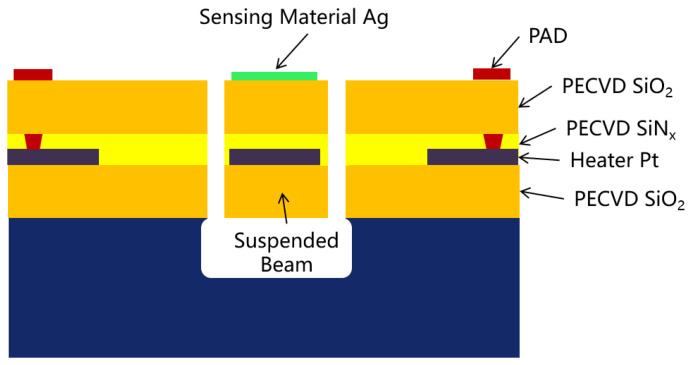
Cross-sectional schematic view of hydrogen sulfide gas sensor with front-side etch.

Figure 2 shows the cross-sectional schematic view of a hydrogen sulfide gas sensor with a front side etch. Silicon dioxide (SiO_2_) and silicon nitride (SiNx) are extensively utilized in the field of microelectronics as commonly employed dielectric and passivation layers. Meanwhile, SiO_2_ layer has compressive stress and SiNx layer has tensile stress. SiO_2_/SiNx/SiO_2_ sandwich structures were selected as the supporting layers which has the advantage of balancing internal stress. The choice of platinum as the microheater material was based on its exceptional stability, linearity, and resistance to oxidation across a broad range of operating temperatures. Silver nano-film acting as a sensing material, because silver has an intrinsically good selectivity for hydrogen sulfide detection and can be deposited by a normal e-beam evaporation process easily, offers perspectives of high consistency and easy mass production. Meanwhile, because the silver metal itself can also be used as a conductive material for the interfinger electrodes, it can be extended to more gas sensor applications after drip coating with other gas-sensitive materials. The design and characterization of this structure also have important guiding significance for other gas sensors. The release of the suspended film is generally performed in two ways: it can be exposed by first depositing all the necessary layers and then etching from the back of the wafer until it reaches the film. This backside etching can be formed with deep reactive ion etching (DRIE). Alternatively, a film may be formed by etching from the top of the prepared opening, a manufacturing step that may be formed using wet chemical or plasma etching. The suspended beam is usually achieved by the second method.

The size effect refers to the phenomenon that, when a material is reduced to the nanometer or micro-nanometer scale, its properties and behavior exhibit distinct deviations from those observed at macroscopic dimensions. In terms of thermal conductivity, the size effect primarily manifests in two aspects: interface scattering resulting from size reduction and quantum confinement effects. Firstly, when the size of the material is reduced to the nanometer level, the proportion of crystal particles, pores, grain boundaries and other interfaces inside the material will increase. These interfaces have an obstructive effect on heat transfer, resulting in reduced thermal conductivity. Therefore, the size effect leads to a decrease in thermal conductivity. Secondly, at the nanoscale, the behavior of electrons and phonons in a material is affected by quantum effects. The scattering process of electrons and phonons is related to the size of the material, resulting in a different heat conduction behavior of the nanomaterial than the macroscopic size. Some experimental studies have shown that the thermal conductivity of materials may show a nonlinear size-dependent relationship at the nanoscale. In general, the size effect leads to a decrease in thermal conductivity; that is, the thermal conductivity of the material at the nano scale is lower than that at the macro scale. This size effect is important for the design and control of thermal management and thermal conductivity of nanomaterials.

According to the microscopic mechanism of heat transport, the mechanism that determines the heat transport process is the interaction between electrons and phonons (metal film) and the scattering of phonons (insulator or semiconductor film). Whether for electrons or phonons, the simple expression of thermal conductivity based on kinetic theory is [23]

(6) $$k=\frac{1}{3}cv\Lambda_{b}$$

For semiconductor materials, *c* is the phonon specific heat capacity, *v* is the mean speed of phonons, and $\Lambda_{b}$ is the mean free path of phonons. For metal, *c* is the electrons specific heat capacity, *v* is the mean speed of electrons, and $\Lambda_{b}$ is the mean free path of electrons. When the thickness of materials is at the nano scale, $\Lambda_{b}$ is determined by thickness and is also influenced by temperature. When the thickness of the material is reduced to close to or much less than $\Lambda_{b}$, the thermal conductivity of the material is very different from that of the bulk. The methods employed for investigating the thermal conductivity of nanofilms encompass experimental measurement, molecular dynamics simulations [24], lattice dynamics calculations [25], and solving the phonon Boltzmann transport equation (BTE) [26,27]. However, experimental measurements are subject to technical limitations and primarily focus on thin films with a thickness exceeding 10 nm.

**Figure 3:**
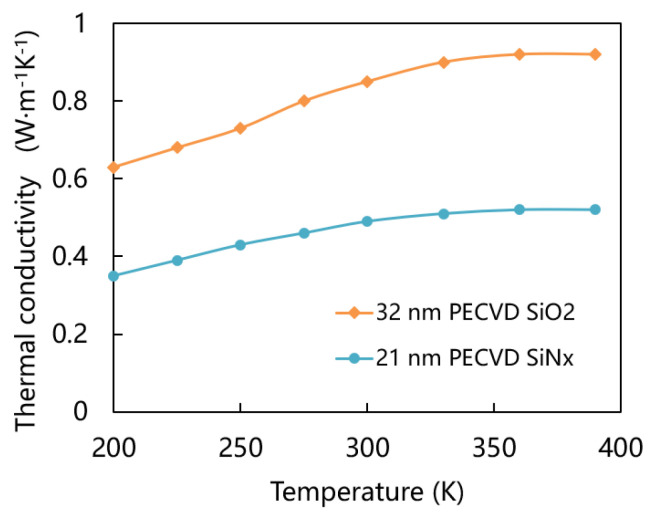
Thermal conductivity of 32 nm thickness SiO_2_ and 21 nm thickness SiNx versus temperature [28].

Lee et al. used the 3-ω method (in brief, the 3-ω method for thin films uses a single metal-line as both the heater and thermometer) to measure the thermal conductivity of SiO_2_ and SiNx films with a thickness of 20–300 nm prepared by PECVD method [28]. For films with a thickness greater than 100 nm, there is little correlation between thermal conductivity and film thickness. Conversely, when the thickness is less than 100 nm, both SiO_2_ and SiNx films exhibit a decrease in thermal conductivity as film thickness decreases. Figure 3 shows the curve of thermal conductivity of 32 nm thickness SiO_2_ and 21 nm thickness SiNx versus temperature. The thermal conductivity of metals has been found to be temperature-independent under moderate and high temperature conditions, as demonstrated by more comprehensive theoretical models and experimental studies [29,30]. When the temperature is close to room temperature, the thermal conductivity changes little with temperature until the melting point is reached. Zhang et al. measured nanosized platinum thin film with 28 nm thickness. The experimental results show that the thermal conductivity of platinum nanofilms is 29.5 W $m^{-1}K^{-1}$ at 25 °C, which is less than half of the bulk value [31]. Similarly, at room temperature, the thermal conductivity of nano silver decreased by 55% compared to bulk [32]. Since the operating temperature of the gas sensor is greater than 200 °C, the thermal conductivity measured by the metal at room temperature according to the previous research has little difference with the thermal conductivity at high temperature, so we use the thermal conductivity of the metal nanofilm at room temperature as the value of the thermal conductivity at high temperature. Silver reacts with hydrogen sulfide to form silver sulfide is an irreversible chemical reaction at room temperature, considering the service life of the gas sensor, we choose 50 nm thickness of silver as the sensing material.

The beam length and shape can be optimized for the lowest power consumption when other parameters are fixed. Table 1 shows the design parameters taking into account lower heat loss and manufacturing considerations. Subsequently, we will employ numerical analysis and finite element simulation to explore the optimization of power consumption.

## 3. Results and Discussion

In order to further support the analysis and calculation, we utilized the finite element simulation software COMSOL Multiphysics 5.6 for parameter verification and conducted simulation analysis on the length, shape, power consumption, stress and displacement at an operating temperature of 200 °C.

**Figure 4:**
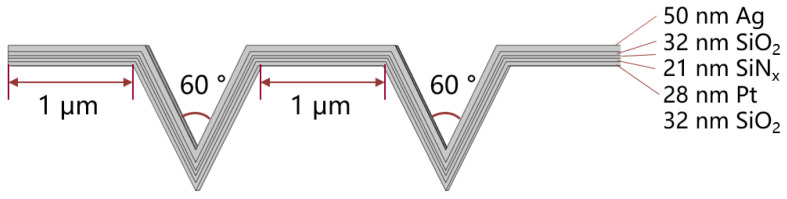
Schematic diagram of the groove width and groove interval of corrugated beam.

**Figure 5:**
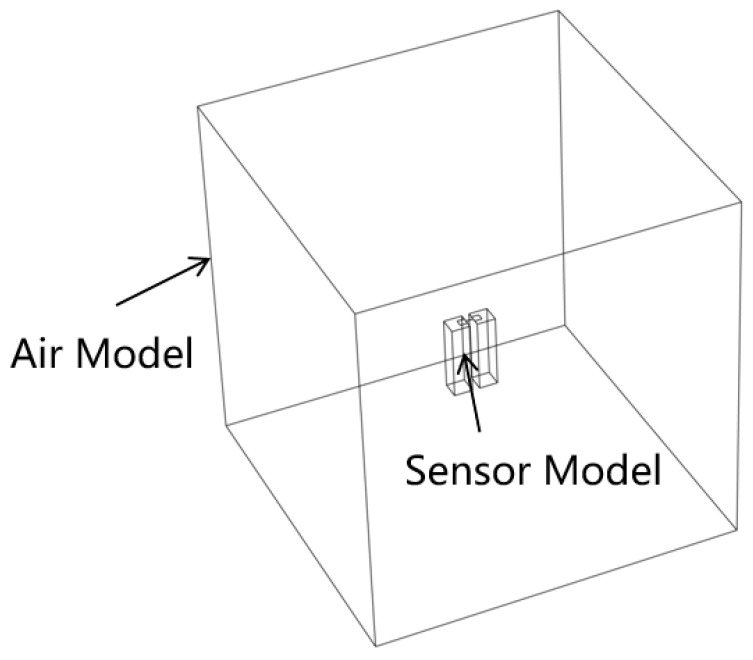
Three-dimensional model of the sensor and air in the software.

Conventional cantilever gas sensors are straight arm, and silver is the gas sensing material can be grown directly by electron beam evaporation, there is no requirement for the shape of the beam. Although corrugated bridge structure has been designed and fabricated in RF MEMS switch [33], there have been few studies focused on this structure in gas sensors. Corrugated beam is a novel shape for a gas sensor, and the impact of this structure on power consumption is worthy of discussion. In order to facilitate verification, we choose a corrugated film with a spacing distance of 1 μm and an equal Angle of 60° V-shape corrugation for simulation as shown in Figure 4. First, we build a three-dimensional model of the sensor in the software. Besides heat transfer from the beam to substrate, heat loss from the beam to the air is very important, so an air model of 1000 μm × 1000 μm × 1000 μm was built, which is much larger than the volume of sensor as shown in Figure 5. Thermal conductivity of metal materials using the values obtained in the previous research, SiO_2_ and SiNx need to manually input the thermal conductivity versus temperature curve. When the temperature is greater than 120 °C, the thermal conductivity is predicted to be little affected by high temperature. Other parameters of the materials were taken from the material library of COMSOL.

The electro-thermal–mechanical simulation generally involves three sets of governing laws: electrostatics, heat transfer statics, and mechanical statics. After defining the geometry, mesh, and material properties, it is essential to simulate the physical behavior of the entire system. The device is initially biased onto the circuit by applying a potential to facilitate current flow and reach the heater. The heater serves as a resistive element that converts electrical energy into heat through joule heating. Optimal materials and shapes must be carefully selected to ensure a uniform distribution of temperature across the active area while minimizing power loss. The temperature rise resulting from Joule heating not only affects the induction film but also elevates the temperature of its constituent material. This thermal variation, along with subsequent cooling, induces mechanical strain when biasing is discontinued in the circuit. Due to different coefficients of thermal expansion (CTE), these materials can undergo mechanical deformation during biasing which may eventually lead to fracture or delamination. We employ Joule heating physics, incorporating its current (ec) and heat transfer in solids (ht) modules to simulate both conduction and convective heat transfer. The Joule heat generated by the voltage applied to the heater is coupled to the solid heat transfer physical field by the following formula [9]:

(7) $$\rho C_{p}\frac{\partial T}{\partial t}+\nabla \left(-k\nabla T\right)=Q$$

where *T* is the temperature, $C_{p}$ is the heat capacity, ρ is the material density, *k* is the thermal conductivity, and *Q* is the heat source. The initial boundary conditions for ambient temperature and gas sensor materials are set at 25 °C. The heat flux convective heat flux equation $q_{0}$ is given as [9]

(8) $$q_{0}=h\left(Text-T\right)$$

and the air convection heat transfer coefficient h is set to 10 W/(m^2^ k). The solid mechanics (solid) and heat transfer in solids (ht) were coupled through Multiple physical field. After determining the temperature distribution, we can utilize the stress and mechanical static equations in conjunction with the solved temperature distribution and thermal expansion coefficient to compute the displacement and all other mechanical parameters [34]:

(9) $$\sigma =E(\varepsilon -\varepsilon_{T})$$

(10) εT=αΔT

where σ is stress, ε is strain, $\varepsilon_{T}$ is initial thermal strain, *E* is Young’s Modulus matrix, α is thermal expansion coefficient, and Δ*T* is differential temperature. The displacement simulation requires at least one fixed point in the simulation space to ensure that only displacements occur rather than deformations of the entire structure. In the finite element simulation, a fixed point is set at the bottom surface of the silicon domain. This calculation provides displacement measurements relative to the bulk wafer.

There are usually some errors between the simulation results and the actual results. The influencing factors of these errors are generally the thermal conductivity difference caused by the film deposition process, such as the crystalline defects, adhesion between layers and the actual film thickness and length differences of the process control. All parameters of the simulation process are regarded as fixed values and the simulation results are not affected by any influencing factors such as the process.

**Figure 6:**
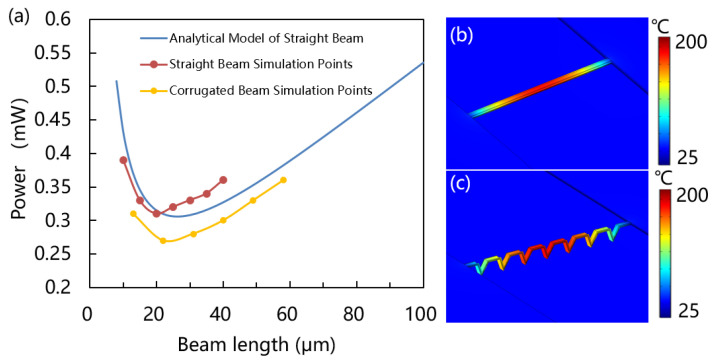
Temperature analysis of straight and corrugated beam. (**a**) Power required to reach 200 °C versus beam length. (**b**) Temperature distribution of 15 μm length straight beam at 200 °C. (**c**) Temperature distribution of 22 μm length corrugated beam at 200 °C.

**Figure 7:**
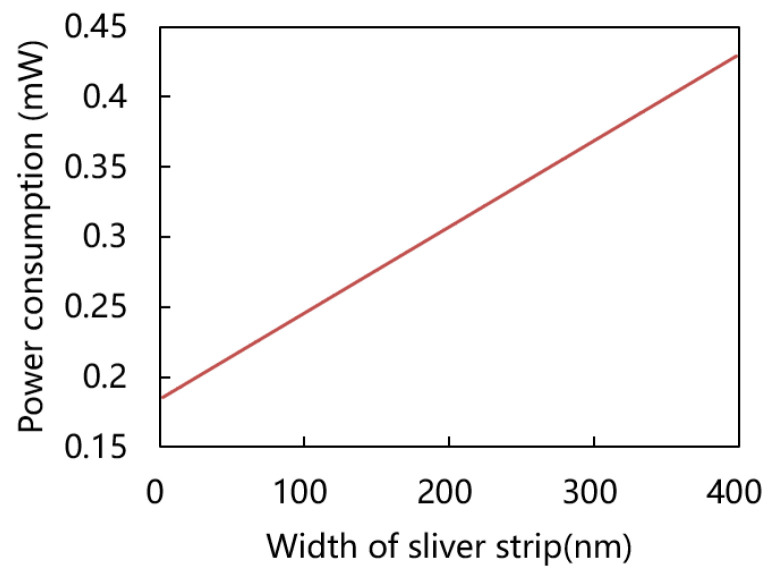
Power consumption at 200 °C versus width of silver strip.

Power consumption is one of the important performances of gas sensors; a higher power consumption indicates need more heat dissipate in order to heat up properly the sensing material that is on top of heater. High power consumption limits the gas sensor application in mobiles and portable devices and the ultra-low power range is usually less than 1 mW. The power consumption of various beam lengths as shown in Figure 6, The minimal power of Matlab numerical analytical model is 0.307 mW when beam length is 26 μm. In order to further support the analytical calculation, the power consumption of straight beam and corrugated beam at 20 °C operating temperature was simulated by finite element method (FEM). The results show the straight beam length at the range of 20 μm and 25 μm has a minimal power of about 0.31 mW and a corrugated beam length at the range of 22 μm and 31 μm has the minimal power about 0.27 mW. The corrugated beam has the lower power consumption may be attributed to the presence of corrugation, which limit heat dissipation in the local space and reduce convective heat loss. Additionally, self-heated corrugation configuration results in reduced overall heat loss at the same power consumption. Through the results, the corrugated structure can also be applied to the design of other shapes of micro-hotplate to reduce heat loss, which provides a certain guiding significance for other applications of micro-hotplate. Figure 6b,c are the temperature distribution of 15 μm length straight beam and 22 μm length corrugated beam at 200 °C operating temperature, respectively. The maximum temperature is in the central region because the heat loss is from the beam center to the substrate. The operating temperature of over 80% of the two structures exceeds 100 °C, thereby satisfying the high-temperature requirements for hydrogen sulfide gas sensors. Since the thermal conductivity of metal silver is many times higher than other materials, the influence of the width of the sensitive material silver on the power consumption is further evaluated as shown in Figure 7. The results show that the power consumption decreases with the decrease in silver width. When the silver width decreases to 100 nm, the power consumption is only 0.245 mW. Compared to 200 nm width power consumption is reduced by 20%. To further reduce power consumption, it may be worth considering reducing the width of the silver.

**Figure 8:**
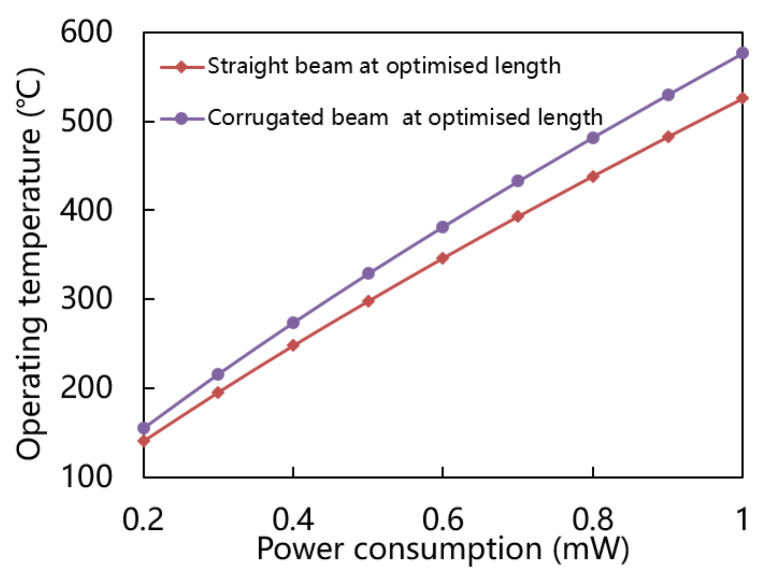
Power consumption versus operating temperature of straight and corrugated beam at optimized length.

Figure 8 shows power consumption versus operating temperature of straight and corrugated beam at various optimized lengths. It is obvious that under the same power consumption, the operating temperature achieved by the corrugated beam is higher than that of the straight beam. Therefore, the corrugated beam has less heat loss. Both structures consume less than 1 mW of power even at operating temperatures of 500 °C.

**Figure 9:**
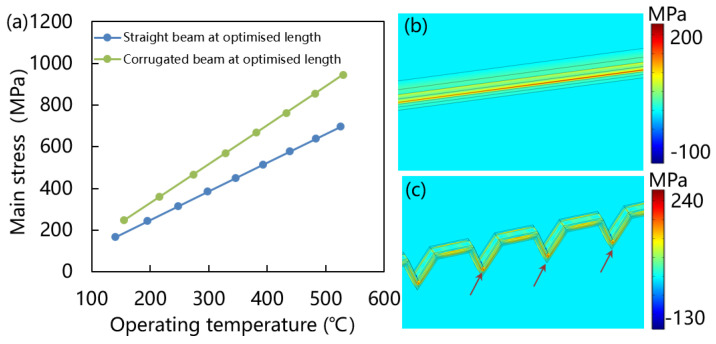
Stress analysis of straight and corrugated beam. (**a**) Main stress versus operating temperature of straight and corrugated beam at optimized length. (**b**) Main stress distribution of 15 μm length straight beam at 200 °C. (**c**) Main stress distribution of 22 μm length corrugated beam at 200 °C.

Stress has an important effect on the thermo-mechanical stability of gas sensors. The post-deposition stress encompasses both inherent and thermal stresses from the multiple layers constituting the film, making it imperative to minimize this stress for ensuring structural stability. When layers are stacked, it is important to have the combined stresses remain below hundreds of megapascals. Figure 9a shows stress analysis of straight and corrugated beam. It can be seen that under the same operating temperature, the main stress of corrugated beam is higher than that of the straight beam. However, the stress values are in the range of several hundred megapascals, which is much lower than that of the full membrane structure. Figure 9b,c are the main stress distribution of 15 μm length straight beam and 22 μm length corrugated beam at 200 °C operating temperature, respectively. The stress concentration points of straight beam are surround the heater at center area, but the stress concentration points of corrugated beam are at the bottom of the V-shaped grooves, which release the stress around the beam membrane. Corrugated structure can effectively release residual stress in the membrane have been confirmed. The residual stress can optimize through adjust the thickness of the diaphragm, the interval and the width of V-shaped grooves [35]. The corrugated film can disperse the central stress and reduce it to the V-shaped grooves.

**Figure 10:**
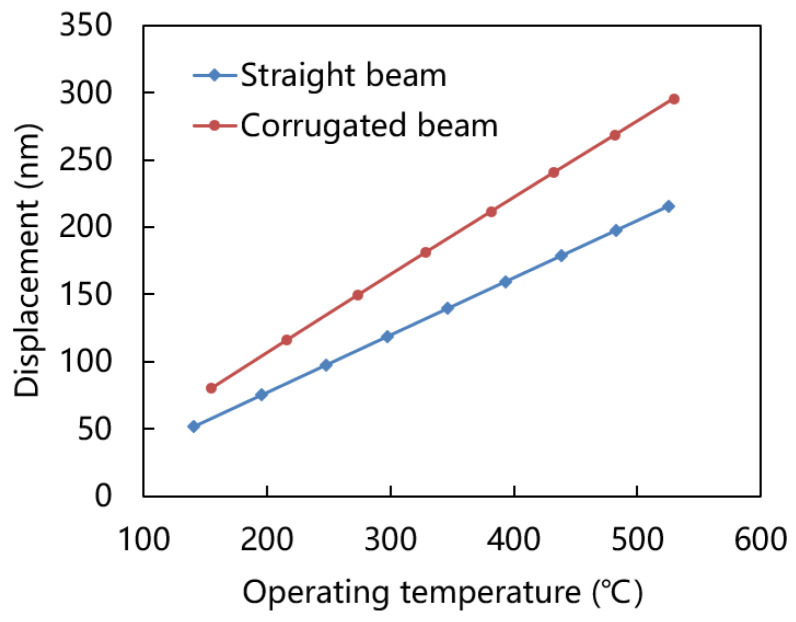
Displacement of straight and corrugated beam at various temperatures.

Figure 10 shows the displacement of straight and corrugated beam at various temperature. Both the straight beam and corrugated beam exhibit a very small displacement deformation (a few hundreds of nanometers) at a wide range operating temperature. These two kinds of structures are not easy to deform and fracture at high temperature, which is very suitable for the application of micro-hotplate.

## 4. Conclusions

In this paper, we proposed a novel corrugated cantilever structure to build an ultra-low-power gas sensor. The power can be reduced through optimizing thermal conductivity of material, the thickness, length and width of the beam. Through numerical analysis and finite element simulation, self-heated corrugation had significantly lower power consumption. Under the operating temperature of 200 °C, only 0.27 mW power is consumed, the stress is less than 250 MPa and the displacement is a few hundreds of nanometers. This design is also useful for other gas sensor applications, and plays a certain guiding significance in the field of MEMS devices which working with power consumption of micro and nano watt.

## Figures and Tables

**Table 1:** Design parameters of suspended beam gas sensor.

	SiO_2_ Layer 1	Pt Layer 2	SiNx Layer 3	SiO_2_ Layer 4	Ag Layer 5
Thickness (nm)	32	28	21	32	50
Width (nm)	400	200	400	400	200

## Data Availability

Data is contained within the article.
